# National and regional trends in the prevalence of type 2 diabetes and associated risk factors among Korean adults, 2009–2021

**DOI:** 10.1038/s41598-023-43353-x

**Published:** 2023-10-04

**Authors:** Jiyeon Oh, Soeun Kim, Myeongcheol Lee, Sang Youl Rhee, Min Seo Kim, Ju-Young Shin, Hyunjung Lim, Seung Won Lee, Masoud Rahmati, Sunyoung Kim, Dong Keon Yon

**Affiliations:** 1 Department of Medicine, Kyung Hee University College of Medicine, Seoul, South Korea; 2 Center for Digital Health, Medical Science Research Institute, Kyung Hee University College of Medicine, Seoul, South Korea; 3 Department of Regulatory Science, Kyung Hee University, Seoul, South Korea; 4 Department of Endocrinology and Metabolism, Kyung Hee University School of Medicine, Seoul, South Korea; 5 Cardiovascular Disease Initiative, Broad Institute of MIT and Harvard, Cambridge, MA USA; 6 School of Pharmacy, Sungkyunkwan University, Suwon, South Korea; 7 Department of Medical Nutrition, Graduate School of East-West Medical Science, Kyung Hee University, Yongin, South Korea; 8 Department of Precision Medicine, Sungkyunkwan University School of Medicine, Suwon, South Korea; 9 Department of Physical Education and Sport Sciences, Faculty of Literature and Human Sciences, Lorestan University, Khoramabad, Iran; 10 Department of Physical Education and Sport Sciences, Faculty of Literature and Humanities, Vali-e-Asr University of Rafsanjan, Rafsanjan, Iran; 11 Department of Family Medicine, Kyung Hee University Medical Center, Kyung Hee University College of Medicine, 23 Kyungheedae-ro, Dongdaemun-gu, Seoul, 02447 South Korea; 12 Department of Pediatrics, Kyung Hee University Medical Center, Kyung Hee University College of Medicine, 23 Kyungheedae-ro, Dongdaemun-gu, Seoul, 02447 South Korea

**Keywords:** Endocrinology, Health care

## Abstract

Disproportionate impact of COVID-19 on socioeconomic and behavioral variables may have impacted the prevalence of diabetes. We utilized nationwide long-term serial study from the 2009 to 2021 Korea Community Health Survey (KCHS). We explored national and regional prevalence and trends of diabetes according to the socioeconomic and behavioral factors before and during the pandemic. Also, we interpreted which groups became more vulnerable to the prevalence of diagnosed diabetes during the pandemic. A total of 2,971,349 adults aged (19 to 39, 40 to 59, and ≥ 60 years) were included in the analysis. The prevalence of diagnosed diabetes increased slowly during the pandemic (11.6% [95% CI 11.5–11.7] in 2020 and 12.4% [95% CI 12.3–12.6] in 2021), compared to the pre-pandemic era (7.9% [95% CI 7.8–7.9] in 2009–2011 and 11.3% [95% CI 11.3–11.4] in 2018–2019). Also, women, low-income group, low-educational group, and infrequent walking group showed less prevalence of diagnosed diabetes than the others. The diabetic population increased slowly than expected during the pandemic. The pandemic seems to contribute to an unanticipated increase in under-diagnosis of diabetes among the already minority. This study may suggest reinforcing access to healthcare services among the minority during the pandemic.

## Introduction

Diabetes poses a public health burden to all nations today. The number of people with diabetes is anticipated to increase from 285 million in 2010 to 700 million by 2045^1^. Those with diabetes are known to have a higher chance of getting life-threatening illnesses, including strokes and heart attacks^2^. Meanwhile, since the emergence of COVID-19 caused by severe acute respiratory syndrome coronavirus 2 (SARS-CoV-2) in Wuhan, China, the COVID-19 pandemic has become a global crisis^3^. Some reports suggest that those who have contracted COVID-19 are more prone to be diagnosed with diabetes than those without COVID-19^4,5^. However, they mostly highlighted pathophysiological associations and they analyzed short-term trend of type 2 diabetes prevalence^4,5^. Since diabetes is caused by multifactorial interactions among social, environmental, and genetic risk factors, it is important to understand the association between the prevalence of diabetes and socioeconomic and behavioral variables during the pandemic^6^. Also, the result of short-term trend may be different from that of long-term trend.

Our hypothesis is that national prevalence of type 2 diabetes could increase, especially during the pandemic. Thus, we aimed to investigate national and regional trends of diabetes according to numerous socioeconomic and associated risk factors, focusing on the prevalence of type 2 diabetes before and during the pandemic. To investigate our hypothesis, we used data from a large-scale and population-based serial study over 13 years (2009 to 2021).

## Methods

### Study population

This study used nationwide data from the 2009 to 2021 Korea Community Health Survey (KCHS)^7^. Korea Disease Control and Prevention Agency (KDCA) has conducted a large population-based survey among adults aged 19 years and older to assess community health promotions since 2008^7^. The questionnaire consists of various topics of health-related behaviors, such as diabetes and obesity. Information on health behaviors, body measurements, and health-related outcomes was collected through interviews with adult household members (aged ≥ 19 years) participating in the study. To maintain proportional representation, the sampling procedure took into account housing type and selected a secondary household through systematic sampling. To produce accurate statistics, population stratification by 253 community health centers was performed^8^. The KCHS data were anonymous, and the study protocol was approved by the KDCA and the Institutional Review Board of Kyung Hee University (KHUH 2022-06-042), participants provided written informed consent. Qualified examiners conducted all health assessments, employing validated techniques and periodically calibrated equipment. This research adhered to the ethical guidelines established by relevant national and institutional review boards for human research and followed the 1975 Helsinki Declaration, as amended in 2008.

2,976,925 adults were asked to participate in KCHS over 13 years. 5576 missing data on height and weight were excluded. Finally, we included a total of 2,971,349 participants at baseline. There were 295,463 patients with type 2 diabetes and 2,675,886 non-diabetic participants.

### Dependent variable

The survey period was the dependent variable. We separated the whole survey period into six- time segments; 2009 to 2011, 2012 to 2014, 2015 to 2017, 2018 to 2019, 2020, and 2021. Considering that the first COVID-19 case in South Korea was reported on January 20, 2020, 2020 was perceived as the early pandemic period and 2021 as the late pandemic period^9^.

### Independent variable

The prevalence of diabetes is the independent variable. Participants were asked to self-report “Have you ever been diagnosed with diabetes by a doctor?”, and were given binary response options for these questions, specifically “yes” or “no”^7^.

### Covariates

Information on covariates was included to eliminate the effects of an additional factor that may distort the actual association. These variables were sex, age (early adulthood [19 to 39] years, middle adulthood [40 to 59 years], and elderly [≥ 60 years]), region of residence (urban and rural)^10^, number of basic livelihood security recipients^11^, household income (unknown, low [< 3 million South Korean won; KRW], middle [3–5 million KRW], and high [≥ 5 million KRW]), education background (high school or less and college or more), occupation (white, blue, and inoccupation), marital status, subjective health level (good, normal, and bad), frequency of walking(< 1, 1–2, 3–4, and ≥ 5 times a week), breakfast eating habits (< 5 and ≥ 5 days a week), body mass index (BMI; underweight, normal, overweight, and obese), frequency of drinking alcohol (hardly drinking, a few times a month, and a few times a week), and smoking status (smoker and non-smoker). The variable of the region of residence was classified into two categories: urban (Seoul, Busan, Daegu, Incheon, Gwangju, Daejeon, Ulsan, Sejong, and Gyeonggi) and rural (Gangwon, Chungbuk, Chungnam, Jeonbuk, Jeonnam, Gyeongbuk, Gyeongnam, and Jeju)^10^.

The variable of occupation was categorized into the following three groups: white (managers, professionals, and clerks), blue (service, sales, agricultural, forestry, fishery, craft, machine operating, elementary workers, and armed forces), and inoccupation (students and housewives), in accordance with to the Korean Standard Classification of Occupations^12^. BMI was calculated based on self-reported height and body weight. The participants were divided into four categories (underweight [< 18.5 kg/m^2^], normal [18.5 to 23 kg/m^2^], overweight [23 to 25 kg/m^2^], and obese [≥ 25 kg/m^2^]) according to the Asia–Pacific criteria of the Western Pacific Regional Office 2000 from the World Health Organization^13^. Basic livelihood security recipients defined as those received by the guarantee of a minimum standard of living and self-reliance for the poor and supported low-income households.

### Statistical analysis

In order to examine the estimates of national prevalence, we performed a weighted complex sampling analysis^14,15^. We used the weighted linear regression models to assess the trend of diabetes rates over the past 13 years, targeting the period amidst the outbreak of COVID-19. Then, a difference of β (β_diff_) was analyzed to explore the trend changes from 2009–2019 to 2019–2021 (before and during the pandemic). Also, we derived the weighted odds ratio (ORs) and 95% confidence interval (CI) from the weighted logistic regression models (2018–2019 to 2020–2021). Sex, education background, region of residence, BMI, income, smoking, alcohol drinking and walking were perceived as covariates in all the linear regression and logistic regression models^16^. BMI was re-classified into two categories: ‘under and normal’ and ‘over and obese’. The frequency of walking variable was re-categorized into three groups (< 1, 1 to 4, and ≥ 5 times/week). Lastly, we obtained the ratio of ORs to estimate the interaction term of each risk factor^17^. We calculated the ratio of ORs for each category using the OR values obtained before and during the pandemic. This ratio allows us to interpret which groups became more vulnerable to the prevalence of diagnosed diabetes during the pandemic^18^.

All the statistical analyses were conducted with R software (version 4.2.2; R Foundation, Vienna, Austria) and Python software (version 3.9.13; Python Software Foundation, Wilmington, Delaware, USA). Testing was two-sided and p-values < 0.05 were considered statistically significant.

## Results

The general characteristics of the participants were given in Table 1. Among the 2,971,349 valid participants, there were 1,344,538 (45.3%) men and 1,626,811 (54.7%) women. Also, 295,463 (9.9%) responded that they were diagnosed with diabetes.

**Table 1:** General characteristics of Korean adults, 2009–2021 (N = 2,971,349).

	Total	Patients with diabetes	Patients without diabetes
Overall, N (%)	2,971,349 (100)	295,463 (9.9)	2,675,886 (90.1)
Sex, n (%)			
Man	1,344,538 (45.3)	143,998 (48.7)	1,200,540 (44.9)
Woman	1,626,811 (54.7)	151,465 (51.3)	1,475,346 (55.1)
Age, years, n (%)			
19–39	757,492 (25.5)	6582 (2.2)	750,910 (28.1)
40–59	1,116,718 (37.6)	78,819 (26.7)	1,037,899 (38.8)
≥ 60	1,097,139 (36.9)	210,062 (71.1)	887,077 (33.2)
Region of residence, n (%)			
Urban	1,425,830 (48.0)	122,721 (41.5)	1,303,109 (48.7)
Rural	1,545,519 (52.0)	172,742 (58.5)	1,372,777 (51.3)
Basic livelihood security recipients, n (%)	106,830 (3.6)	20,525 (6.9)	86,305 (3.2)
Income, n (%)			
Unknown	252,554 (8.5)	23,999 (8.1)	228,555 (8.5)
Low	1,476,653 (49.7)	195,930 (66.3)	1,280,723 (47.9)
Middle	710,531 (23.9)	45,883 (15.5)	664,648 (24.8)
High	531,611 (17.9)	29,651 (10.0)	501,960 (18.8)
Education background, n (%)			
High school or less	1,936,757 (65.2)	254,827 (86.2)	1,681,930 (62.9)
College or more	1,034,592 (34.8)	40,636 (13.8)	993,956 (37.1)
Occupation category, n (%)			
White	566,364 (19.1)	22,312 (7.6)	544,052 (20.3)
Blue	1,445,590 (48.7)	146,203 (49.5)	1,299,387 (48.6)
Inoccupation	959,395 (32.3)	126,948(43.0)	83,247 (31.1)
Marital status, yes, n (%)	2,089,472 (70.3)	215,286 (72.9)	1,874,186 (70.0)
Subjective health level, n (%)			
Good	1,154,883(38.9)	45,465 (15.4)	1,109,418 (41.5)
Normal	1,216,849 (41.0)	108,657 (36.8)	1,108,192 (41.4)
Bad	599,617 (20.2)	141,341 (47.8)	458,276 (17.1)
Frequency of walking, n (%)			
< 1 times/week	676,219 (22.8)	79,302 (26.8)	596,917 (22.3)
1–2 times/week	347,129 (11.7)	28,696 (9.7)	318,433 (11.9)
3–4 times/week	463,835 (15.6)	44,351 (15.0)	419,484 (15.7)
≥ 5 times/week	1,484,166 (49.9)	143,114 (48.4)	1,341,052 (50.1)
Eating breakfast, n (%)			
< 5 days/week	734,631 (24.7)	28,145 (9.5)	706,486 (26.4)
≥ 5 days/week	2,236,718 (75.3)	267,318 (90.5)	1,969,400 (73.6)
BMI, n (%)			
Underweight	251,832 (8.5)	25,306 (8.6)	226,526 (8.5)
Normal	1,241,274 (41.8)	90,929 (30.8)	1,150,345 (43.0)
Overweight	697,093 (23.5)	74,282 (25.1)	622,811 (23.3)
Obese	781,150 (26.3)	104,946 (35.5)	676,204 (25.3)
Smoking status, n (%)			
Smoker	569,847 (19.2)	50,503 (17.1)	519,344 (19.4)
Non-smoker	2,401,502 (80.8)	244,960 (82.9)	2,156,542 (80.6)
Alcohol consumption, n (%)			
Hardly drinking	983,370 (33.1)	142,703 (48.3)	840,667 (31.4)
A few times a month	142,703 (44.3)	98,583 (33.4)	1,217,573 (45.5)
A few times a week	840,667 (22.6)	54,177 (18.3)	617,646 (23.1)

*BMI* body mass index, *CI* confidence interval.

Table 2 presents the prevalence of diagnosed diabetes according to risk factor groups over 13 years and its trend before and during the COVID-19 pandemic. The number of people with diabetes consistently rises from 7.9% in 2009 to 2011 to 12.4% in 2021. However, the prevalence of diabetes has been increasing slowly during the pandemic, compared to the pre-pandemic era (β_diff_, − 0.76; 95% CI − 1.34 to − 0.18). The same results were observed regardless of sex, region of residence, and smoking status. Also, the low-education (β_diff_, − 1.07; 95% CI − 1.85 to − 0.29), overweight and obese (β_diff_, − 0.70; 95% CI − 1.39 to − 0.01), low-income (β_diff_, − 1.77; 95% CI − 2.68 to − 0.86), and infrequent walking (< 1 times/week [β_diff_, − 1.46; 95% CI − 2.67 to − 0.25] and 1–4 times/week [β_diff_, − 1.00; 95% CI − 1.78 to − 0.22]) groups.

**Table 2:** National trend of the prevalence of diagnosed diabetes before and during the COVID-19 pandemic, weighted % (95% CI).

	Pre-pandemic				During the pandemic		Trend of the pre-pandemic era, β	Trend of the pandemic era, β	Trend difference, β diff (95% CI)	Weighted odds of before and during the pandemic, OR
	2009–2011	2012–2014	2015–2017	2018–2019	2020	2021				2020–2021 to 2018–2019
Overall	7.9 (7.8 to 7.9)	9.2 (9.1 to 9.2)	10.5 (10.4 to 10.6)	11.3 (11.3 to 11.4)	11.6 (11.5 to 11.7)	12.4 (12.3 to 12.6)	1.15 (0.78 to 1.52)	0.39 (– 0.06 to 0.84)	** − 0.76 (− 1.34 to − 0.18)**	**1.067 (1.053 to 1.080)**
Sex, weighted % (95% CI)										
Men	8.3 (8.2 to 8.4)	9.8 (9.7 to 9.9)	11.4 (11.3 to 11.5)	12.3 (12.2 to 12.4)	12.7 (12.5 to 12.9)	13.7 (13.5 to 13.9)	1.36 (0.89 to 1.84)	0.52 (0.01 to 1.03)	** − 0.84 (− 1.54 to − 0.14)**	**1.084 (1.064 to 1.104)**
Women	7.5 (7.4 to 7.6)	8.7 (8.6 to 8.7)	9.8 (9.7 to 9.9)	10.6 (10.4 to 10.7)	10.6 (10.4 to 10.8)	11.4 (11.2 to 11.6)	1.04 (0.76 to 1.32)	0.29 (-0.23 to 0.81)	** − 0.75 (− 1.34 to − 0.16)**	**1.047 (1.028 to 1.066)**
Education background, weighted % (95% CI)										
High school or less	10.2 (10.1 to 10.2)	12.1 (12.0 to 12.2)	14.1 (14.0 to 14.2)	15.2 (15.0 to 15.3)	15.8 (15.6 to 16.0)	16.9 (16.7 to 17.1)	1.70 (1.08 to 2.32)	0.63 (0.15 to 1.11)	** − 1.07 (− 1.85 to − 0.29)**	**1.091 (1.075 to 1.106)**
College or more	2.9 (2.9 to 3.0)	3.4 (3.3 to 3.4)	4.1 (4.0 to 4.2)	4.5 (4.4 to 4.6)	4.9 (4.7 to 5.0)	5.5 (5.3 to 5.6)	0.55 (0.38 to 0.72)	0.39 (0.17 to 0.61)	− 0.16 (– 0.44 to 0.12)	**1.167 (1.130 to 1.204)**
Region of residence, weighted % (95% CI)										
Rural	8.7 (8.6 to 8.8)	10.3 (10.2 to 10.4)	11.9 (11.8 to 12.0)	12.8 (21.6 to 12.9)	13.1 (12.9 to 13.3)	14.1 (13.9 to 14.3)	1.39 (0.87 to 1.91)	0.49 (− 0.03 to 1.01)	** − 0.90 (− 1.64 to − 0.16)**	**1.076 (1.058 to 1.094)**
Urban	7.0 (6.9 to 7.1)	7.9 (7.8 to 8.0)	9.0 (8.9 to 9.0)	9.8 (9.7 to 9.9)	10.0 (9.9 to 10.2)	10.6 (10.4 to 10.8)	0.95 (0.78 to 1.12)	0.30 (− 0.03 to 0.63)	** − 0.65 (− 1.02 to − 0.28)**	**1.058 (1.038 to 1.079)**
BMI, weighted % (95% CI)										
Under and normal	6.2 (6.1 to 6.2)	7.4 (7.3 to 7.5)	8.4 (8.4 to 8.5)	8.8 (8.7 to 8.9)	8.9 (8.7 to 9.1)	9.8 (9.6 to 9.9)	0.88 (0.32 to 1.44)	0.27 (− 0.61 to 1.15)	− 0.61 (− 1.65 to 0.43)	**1.066 (1.044 to 1.089)**
Over and obese	9.9 (9.8 to 10.0)	11.2 (11.1 to 11.3)	12.6 (12.5 to 12.7)	13.5 (13.3 to 13.6)	13.8 (13.6 to 14.0)	14.7 (14.5 to 14.9)	1.20 (0.89 to 1.55)	0.50 (− 0.11 to 1.11)	** − 0.70 (− 1.39 to − 0.01)**	**1.068 (1.051 to 1.086)**
Income, weighted % (95% CI)										
Unknown	8.0 (7.7 to 8.2)	9.3 (8.9 to 9.7)	9.5 (8.8 to 10.2)	9.7 (9.5 to 10.0)	9.8 (9.5 to 10.0)	10.4 (10.2 to 10.7)	0.53 (− 0.29 to 1.35)	0.21 (− 0.19 to 0.61)	− 0.32 (− 1.23 to 0.59)	**1.040 (1.008 to 1.074)**
Low	9.9 (9.8 to 10.0)	12.2 (12.1 to 12.4)	14.1 (14.0 to 14.2)	16.8 (16.7 to 17.0)	17.0 (16.7 to 17.2)	18.2 (17.9 to 18.4)	2.26 (1.80 to 2.72)	0.49 (− 0.29 to 1.27)	** − 1.77 (− 2.68 to − 0.86)**	**1.052 (1.034 to 1.071)**
Middle	4.9 (4.8 to 5.0)	5.6 (5.5 to 5.7)	6.4 (6.3 to 6.5)	8.2 (8.0 to 8.3)	8.8 (8.5 to 9.0)	9.6 (9.4 to 9.9)	1.07 (0.27 to 1.87)	0.56 (0.19 to 0.93)	− 0.51 (− 1.39 to 0.37)	**1.141 (1.104 to 1.179)**
High	4.7 (4.5 to 4.8)	5.0 (4.8 to 5.1)	5.7 (5.5 to 5.8)	5.9 (5.8 to 6.1)	6.3 (6.1 to 6.5)	7.1 (6.9 to 7.3)	0.43 (0.15 to 0.71)	0.41 (0.01 to 0.81)	− 0.02 (− 0.51 to 0.47)	**1.142 (1.102 to 1.184)**
Smoking, weighted % (95% CI)										
Smoker	6.9 (6.8 to 7.1)	8.0 (7.9 to 8.2)	9.4 (9.3 to 9.6)	10.4 (10.2 to 10.7)	11.2 (10.9 to 11.6)	12.4 (12.1 to 12.7)	1.19 (0.97 to 1.41)	0.75 (0.25 to 1.25)	** − 0.44 (− 0.99 to 0.11)**	**1.149 (1.112 to 1.186)**
Non-smoker	8.2 (8.1 to 8.2)	9.4 (9.4 to 9.5)	10.7 (10.7 to 10.8)	11.5 (11.4 to 11.6)	11.7 (11.5 to 11.8)	12.4 (12.3 to 12.6)	1.12 (0.79 to 1.45)	0.30 (− 0.11 tot 0.71)	** − 0.82 (− 1.35 to − 0.29)**	**1.051 (1.037 to 1.066)**
Walking, weighted % (95% CI)										
< 1 times/week	8.8 (8.6 to 8.9)	10.6 (10.5 to 10.8)	12.7 (12.6 to 12.9)	14.0 (13.8 to 14.2)	13.6 (13.3 to 13.9)	15.0 (14.7 to 15.3)	1.77 (1.29 to 2.25)	0.31 (− 0.80 to 1.42)	** − 1.46 (− 2.67 to − 0.25)**	1.023 (0.998 to 1.050)
1–4 times/week	7.1 (7.0 to 7.2)	8.1 (8.0 to 8.3)	9.3 (9.2 to 9.5)	10.5 (10.4 to 10.7)	10.2 (10.0 to 10.5)	11.1 (10.8 to 11.3)	1.14 (0.99 to 1.29)	0.14 (− 0.63 to 0.91)	** − 1.00 (− 1.78 to − 0.22)**	1.012 (0.987 to 1.037)
≥ 5 times/week	7.9 (7.8 to 8.0)	9.0 (8.9 to 9.1)	10.1 (10.0 to 10.2)	10.7 (10.6 to 10.8)	11.4 (11.2 to 11.6)	12.2 (12.0 to 12.4)	0.95 (0.58 to 1.32)	0.56 (0.28 to 0.84)	− 0.39 (− 0.85 to 0.07)	**1.118 (1.098 to 1.139)**

*BMI* body mass index, *CI* confidence interval.

Numbers in bold indicate a significant difference (*P* < 0.05).

**Figure 1:**
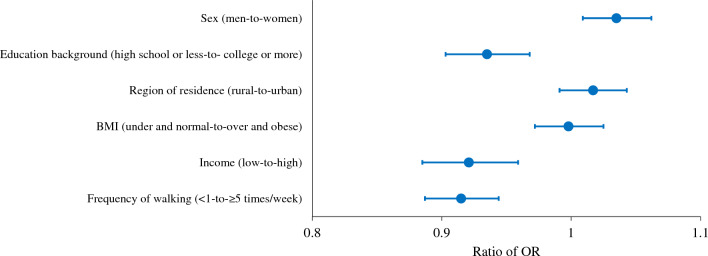
Ratio of ORs plot for association between the prevalence of diabetes and each socioeconomic and behavioral factor including sex, education background, region of residence, BMI, income, and frequency of walking. Blue dots indicate ratio of ORs; Error bars indicate 95% CIs. *BMI* body mass index, *CI* confidence interval, *OR* odds ratio.

To strengthen the hypothesis, we derived the ratio of OR of the prevalence of diagnosed diabetes before and during the pandemic and demonstrated in Fig. 1 and Table 3. Comparing the pre-pandemic and pandemic periods, men had a higher odds ratio of 8.4% (OR, 1.084; 95% CI 1.064 to 1.104) during the pandemic, while women had a lower increase in their odds ratio of 4.7% (OR, 1.047; 95% CI 1.028 to 1.066) then increase of men. To further clarify this evidence, we calculated the ratio of odds ratios, and indeed we found that that the prevalence of diabetes increased more significantly among men than women (ratio of OR, 1.035; 95% CI 1.009 to 1.062). In addition, high level of education (ratio of OR, 0.935; 95% CI 0.903 to 0.968), high income (ratio of OR, 0.921; 95% CI 0.885 to 0.959), and frequent walking (ratio of OR, 0.915; 95% CI 0.887 to 0.944) groups showed a higher prevalence of diagnosed diabetes during the pandemic compared with other groups.

**Table 3:** Ratio of ORs for association between the prevalence of diabetes and each socioeconomic and behavioral factor.

Risk factor	Ratio of OR (95% CI)	Significant direction
Sex (men-to-women)	**1.035 (1.009 to 1.062)**	**Men**
Education background (high school or less-to- college or more)	**0.935 (0.903 to 0.968)**	**College or more**
Region of residence (rural-to-urban)	1.017 (0.991 to 1.043)	None
BMI (under and normal-to-over and obese)	0.998 (0.972 to 1.025)	None
Income (low-to-high)	**0.921 (0.885 to 0.959)**	**High income**
Frequency of walking (< 1-to- ≥ 5 times/week)	**0.915 (0.887 to 0.944)**	** ≥ 5 times/week**

*BMI* body mass index, *CI* confidence interval, *OR* odds ratio.

Bold numbers indicate a significant difference (*P* < 0.05).

**Figure 2:**
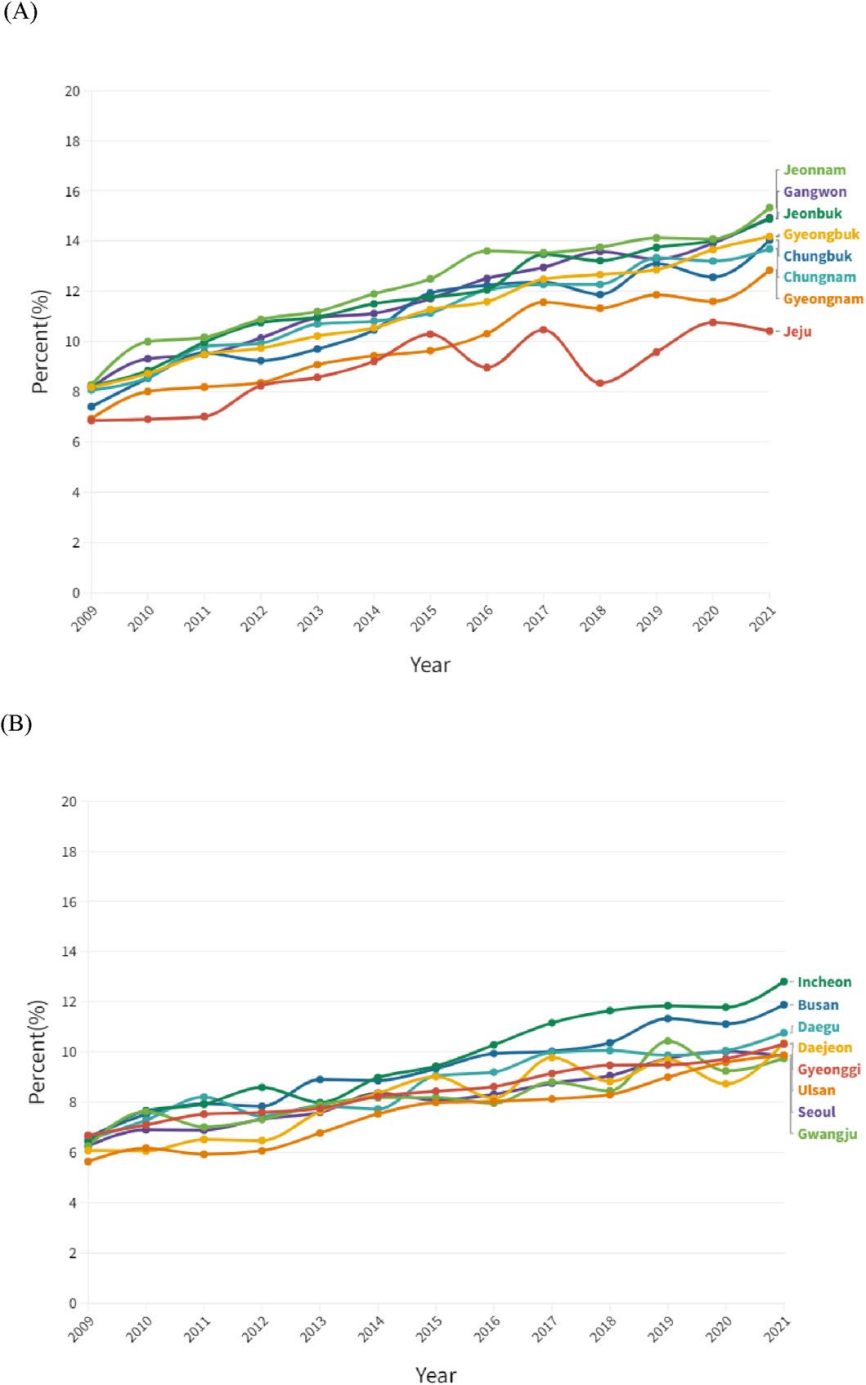
Regional trend of the prevalence of diabetes amongst (**A**) rural and (**B**) urban regions, 2009 to 2021.

**Figure 3:**
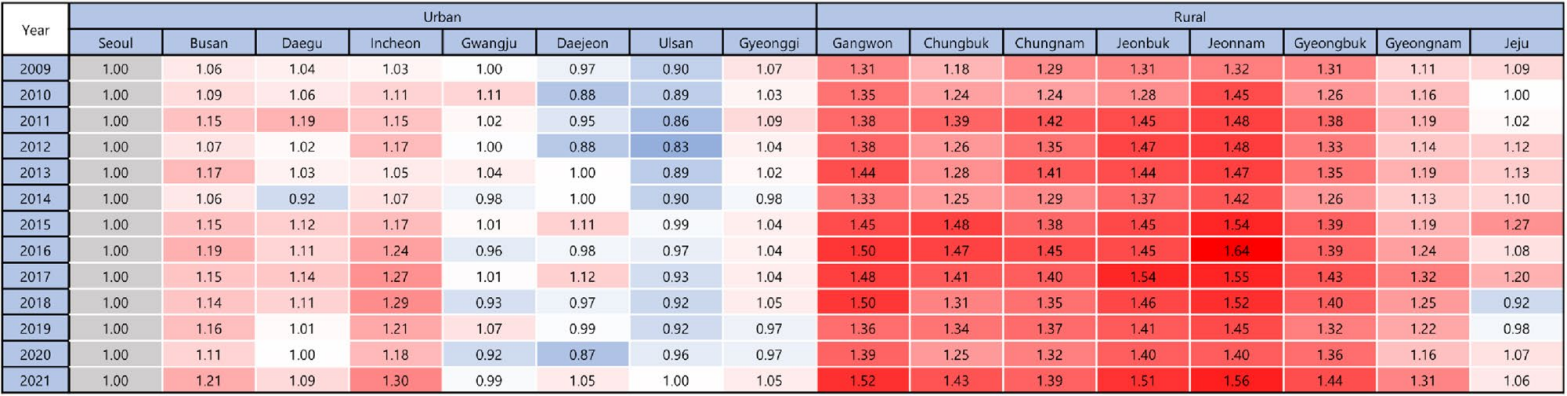
Regional relative prevalence ratio of diagnosed diabetes in urban and rural regions (reference: Seoul), 2009–2021.

**Figure 4:**
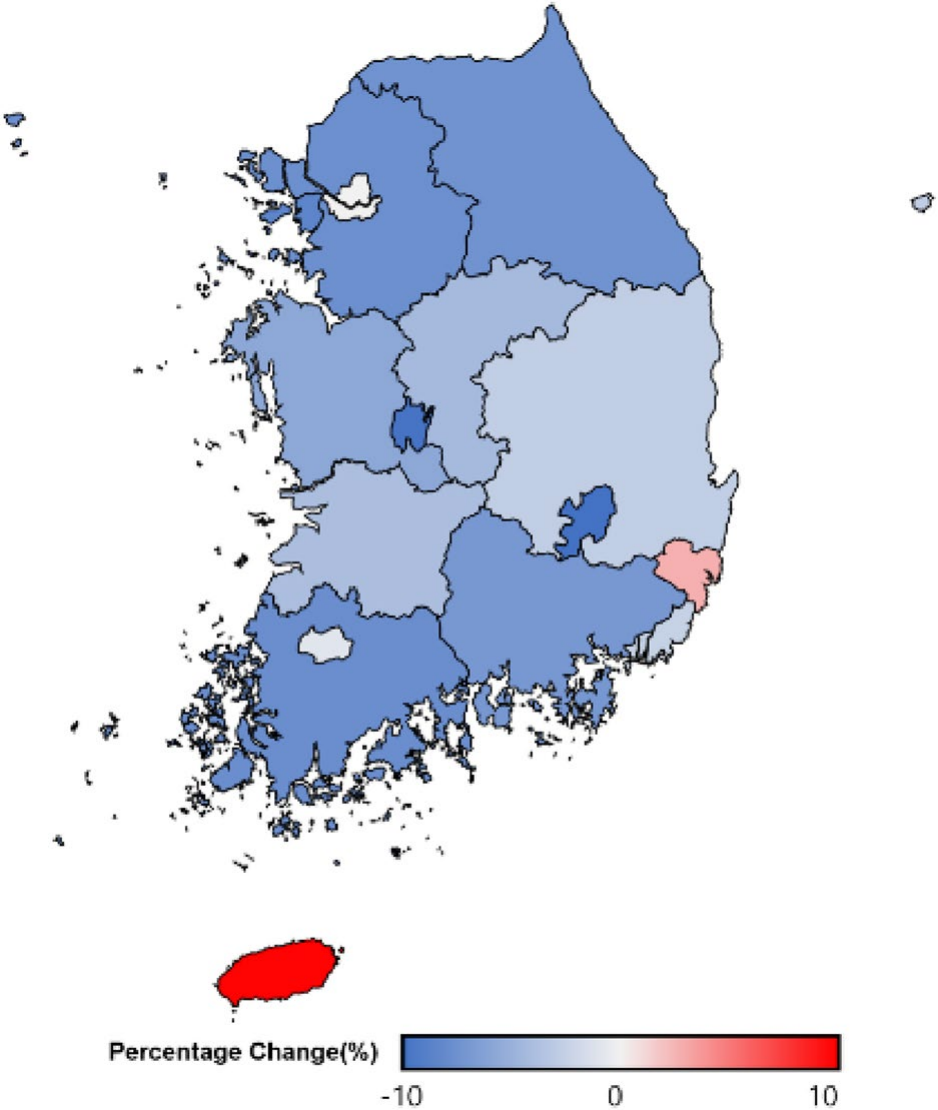
Regional difference of the diabetes prevalence before and during the pandemic in South Korea, 2018–2019 versus 2020–2021.

Figures 2, 3 and 4 indicate regional prevalence of diagnosed diabetes. All rural regions show considerably higher prevalence of diabetes than urban regions. Nevertheless, the prevalence of all regions is presented the tendency of positive slope.

## Discussion

### Key findings

To the best of our knowledge, this is the first large-scale study describing national and regional 13-year trends of the prevalence of type 2 diabetes in South Korea. We aimed to explore the trend difference in the diabetic population amidst the outbreak of COVID-19 and the factors associated with the prevalence. The findings of the study highlight that the diabetic population increased from 7.9% in 2009 to 2011 to 12.4% in 2021. The degree of increase in the number of people with diabetes has been slowed down during the pandemic. Furthermore, the prevalence of diagnosed diabetes differed substantially across socioeconomic subgroups during the pandemic, compared to the pre-pandemic era. During the pandemic women, those from low household income, low educational achievement and infrequent walking habits groups may have been underdiagnosed with type 2 diabetes due to disproportionate impact of COVID-19.

### Comparison with previous studies

The results of this study align with previous studies; an increased prevalence of diabetes was observed due to a lack of access to medical care and preventive medicine in the pandemic era^6,19^. However, since they only analyzed the short-term trend of diabetes prevalence, they did not identify that during the pandemic the prevalence of diabetes elevated less than expected. We concluded that the prevalence of diabetes increased slowly during the pandemic than expected.

In addition, prior studies demonstrated how COVID-19 impacted on diabetes and vice versa. However, its association mostly focused on pathology^20^, not socioeconomic factors. Since diabetes is caused by the multifactorial interplay among social, environmental, and genetic factors, there is a need to investigate the association between the prevalence of diabetes and variables at the individual and social levels before and during the pandemic.

### Possible mechanisms

This study showed a significant deceleration in the total number of patients with diagnosed diabetes during the pandemic. It may be driven by the reluctance of hospital visits. Some previous studies noted that 41% reported having avoided medical care due to concerns about COVID-19^21^. If this tendency were to be maintained for a long time, people would lose chances to manage chronic diseases and detect new conditions, which may aggravate health outcomes.

Also, during the pandemic, the number of people diagnosed with diabetes is fewer than expected among underprivileged individuals. We speculate that the actual population suffering from diabetes is slightly different from those diagnosed with diabetes, especially during the pandemic. The pandemic has magnified disparities in access to health services and lack of control over the allocation of health resources. The already vulnerable communities had difficulties in access to physician consultation, although telemedicine was temporarily allowed in South Korea^22^. Lower income and lower education level groups are prone to have lower perception and acceptance of digital healthcare^23,24^. Likewise, a previous study suggests that the pandemic triggered an increase in delayed disease diagnosis among racial and ethnic minorities in the US^25^. Thus, the pandemic may have contributed to further rises in the under-diagnosis of diabetes among the already vulnerable groups^26^.

### Policy implications

We interpreted that low-income, low educational levels, high BMI, and infrequent walking groups have under-diagnosed diabetes since the outbreak of COVID-19, compared with the pre-pandemic era. Since some studies reported that early detection of diabetes decreases cardiovascular morbidity and mortality^27^, the importance of early diagnosis of diabetes cannot be ignored. A sustainable response is needed at the policy level, such as providing inclusive telemedicine and home health services for those who have prediabetes. It is reported that remote monitoring glucose levels enhances the control of diabetes^28^.

### Strengths and limitations

While the findings of the study revealed how sociodemographic and health-related factors relate to the prevalence of diabetes, this study has some limitations. First, self-reported history of diabetes diagnosis was used in this study, which may recall response bias. However, some studies indicated that self-reported diagnosis history showed good agreement with the actual medical records^29^. Second, since the survey was conducted exclusively for Koreans, the result reflects the sociocultural context of Korea. It may be different from worldwide tendency. Third, we cannot reflect the association between SARS-CoV-2 infection and diabetes mellitus because we have no information on the participants’ SARS-CoV-2 infection history. Forth, due to the lack of questions, we could not collect subsidiary information on diabetes such as family history and the latest diabetes treatment. Finally, we did not inspect under-diagnosis with diabetes among the participants; under-diagnosis is one of the plausible mechanisms to explain the results of this study.

Despite several limitations, this is the first study investigating a 13-year trend in the prevalence of diabetes among Korean adults, and examining the variables at the individual and social level associated with the risk of diabetes. We analyzed 13 consecutive years of data including 2,971,349 participants selected by weighted stratified sampling, which represents the whole population of Korea. The findings of the study may suggest eliminating barriers to healthcare among vulnerable groups during the pandemic.

## Conclusions

This study examined trends in the prevalence of type 2 diabetes in the Korean population from 2009 to 2021 and the associations between the prevalence of diabetes and each risk factor before and during the pandemic. During the pandemic, the prevalence of diagnosed diabetes increased slowly compared to the pre-pandemic era. Moreover, a lower occurrence of diagnosed diabetes was observed in men, those with high-income, high-level education groups, and those with frequent walking habits during the pandemic. The pandemic seemed to attenuate access to healthcare and an unprecedented increase in under-diagnosis among the minority.

## Data Availability

Data are available on reasonable request. Study protocol, statistical code: available from DKY (email: yonkkang@gmail.com). Data set: available from the Korea Disease Control and Prevention Agency (KDCA) through a data use agreement.
